# The influence of shame on posttrauma disorders: have we failed to see the obvious?

**DOI:** 10.3402/ejpt.v6.28847

**Published:** 2015-09-22

**Authors:** Terry F. Taylor

**Affiliations:** School of Psychology, Social Work and Social Policy, University of South Australia, Adelaide, Australia

**Keywords:** PTSD, shame, maladaptive shame regulation, anger, substance abuse, depression

## Abstract

**Background:**

While fear is known to be the dominant affect associated with posttraumatic stress disorder (PTSD), the presence and possible influence of other emotions is less well explored. Recent changes to diagnostic criteria have added anger, guilt and shame alongside fear as significant emotional states associated with the disorder. This article suggests that shame is a frequent, often poorly recognised sequel to trauma, occurring as a result of the meaning the individual places on the traumatic experience and on subsequent interpersonal and environmental events.

**Methods:**

The article reviews the literature on the socio-interpersonal aspects of the posttraumatic experience with particular emphasis on the emotion of shame as both primary and secondary emotion, in its intrapersonal and interpersonal contexts, and in adaptive and maladaptive forms.

**Results:**

The review suggests that posttrauma shame, and maladaptive shame regulation strategies, often manifesting as anger, substance abuse, social withdrawal or depression, may play an important role in the maintenance or exacerbation of the symptoms of PTSD and the development of co-morbidities.

**Conclusion:**

The recognition of shame and maladaptive shame regulation strategies in PTSD treatment and management is critical. However, because shame is frequently considered a painful and discomforting emotion, it may fail to be addressed in the therapeutic setting by both client and therapist. Examination of potential shame-related changes in self-concept, close interpersonal relationships and social inclusion are recommended for individuals who have experienced a range of traumas to identify and address any underlying unacknowledged shame.

For the first time, the diagnostic criteria for posttraumatic stress disorder (PTSD) in the Diagnostic and Statistical Manual of the American Psychiatric Association, 5th Edition (DSM-5) (American Psychiatric Association [APA], 2013), have included persistent negative emotional states of fear, horror, anger, guilt, or shame. Whether, and how, these emotional states might influence the course of the disorder has received limited coverage in the existing literature.

This article explores theories of shame and maladaptive shame regulation, and the role these might play in the exacerbation and perpetuation of posttrauma disorders. It examines the literature on trauma-related shame. It discusses its role as a primary affect occurring in the peri-traumatic period, and as a secondary emotion following appraisal. It further defines its intrapersonal and interpersonal manifestations and their interactions, its connection with neurobiological processes and the importance of its recognition in treatment and management.

## Shame in everyday life

Shame in Western culture is considered a virtually invisible, ubiquitous part of everyday life by Scheff (2014); associated with feelings of weakness, vulnerability, and the likelihood of rejection (Lansky, 2003); and hidden, because it is shameful in itself (Kaufman, 1989). Because the experience of shame is often considered to be painful and disempowering, and because recognition of shame in itself can be felt as shameful, it has been suggested that it may evoke any one, or a combination of, maladaptive shame regulation strategies or defences (Elison, 2005; Elison, Garofolo, & Velotti, 2014; Nathanson, 1987, 1992; Velotti, Elison, & Garofolo, 2014; Webb, 2003, 2010). These reactions are consistent with many of the symptoms and co-morbidities of PTSD. They include anger and violence, substance addiction and isolation (Van der Kolk, 2013), and the often-accompanying feelings of hopelessness and helplessness that can progress to depression, and ultimately to suicide (Violanti, Andrew, Mnatsakanova, Hartley, Fekedulegn, & Burchfiel, 2015).

## Shame and guilt: distinguishing between the two emotions

There are differing contemporary theoretical accounts of the nature of shame and guilt. One group of authors represented by M. Lewis (2003), Tangney and Dearing (2002), and Tracy and Robins (2004), consider shame a destructive emotion with little or no adaptive value, and guilt the adaptive and mature emotion. These authors, some of whom have received prominence as a result of their authorship of the Test of Self Conscious Affect (Tangney, Wagner, & Gramzow, 1989), consider both guilt and shame to be “self-conscious emotions”—a product of evaluation of one's behaviour or one's “self” with reference to a particular standard. This is said to require a cognitive capacity that is not achieved before the age of 2 or 3 years (M. Lewis, 2000), implying that shame cannot exist as an affect before that age. However considerable evidence exists for the observation of shame in much younger children than this (Izard, 1971; Nathanson, 1992; Tomkins, 1962, 1963) suggesting that the affect of shame is not contingent on level of cognitive development.

A different view of shame is held by these affect theorists. First described by Charles Darwin in the late 19th century, primary or basic affects are considered to be universal, found from infancy onwards in cultures worldwide, with common facial expressions and postural characteristics (Darwin, 1872/1965; Ekman, 1972, 1973; Tomkins, 1962). The spectra of primary affects (with some minor variations across theories) include embarrassment–shame–humiliation; irritability–anger–rage; sadness–distress–grief; interest–excitement–awe; surprise–startle–shock; anxiety–fear–terror; happiness–joy–rapture; and disgust/dissmell (Ekman, 1972; Nathanson, 1987, 1989; Tomkins, 1962, 1963, 1991, 1992; Webb, 2003). In the terminology of affect theory, *feelings* occur as a result of awareness of the *affects* (Basch, 1976) and *emotions* are composed of the feelings, together with cognitive associations with, and behavioural reactions to, previous experience of them—the autobiographical memory (Nathanson, 1992).

Within affect theory, shame has an observable characteristic set of facial and postural signs. They include the breaking of eye contact, the lowering and turning away of the face, upper body slump, and dilation of blood vessels of the face and neck (Darwin, 1872/1965; Nathanson, 1992; Webb, 2003). There are parallels to the physiological characteristics of shame in the animal world in the dominance/submission behaviours of primates, thought to serve an evolutionary purpose in averting intra-species attack (Keltner & Harker, 1998). There are recognisable cross-cultural and historical variations in the function of shame. In parts of Asia, Africa, and South and Central America, and in collectivist cultures generally, it is often valued as serving an adaptive function regulating social behaviour; here the display of shame is considered positive (Sheikh, 2014).

## Peri-traumatic shame

When viewed as a sequel to trauma, shame may potentially manifest as a *primary* emotion, occurring as a peri-traumatic reaction at the time of the traumatic exposure or as a *secondary* emotion via the process of subsequent cognitive appraisal of the meaning and its future implications, or as both.

Ozer, Best, Lipsey, and Weiss (2003) in a meta-analysis of associated empirical research, found that the intensity of the peri-traumatic emotions (in which they included fear, helplessness, horror, guilt, and shame) was among the strongest correlates of PTSD, and that higher distress in the peri-traumatic period was related to higher level of symptoms. In an analysis of trauma clinic patients, Holmes, Grey, and Young (2005) examined the intrusive memories and emotionally charged “hotspots” of trauma memories. They found that these memories, which were considered to reflect peri-traumatic processing, more often related to a severe negative view of the self than to fear, helplessness, or horror, and emphasised that this needed to be considered in the treatment of PTSD. This severe negative view of the self corresponds to shame, suggesting that shame could be strongly implicated in the peri-traumatic response.

## Shame as a secondary emotion

Secondary emotions were considered by Brewin, Andrews, and Rose (2000) to be fundamentally different from primary emotions as they are based on cognitive appraisals following the trauma and may have an important impact on the later development of PTSD. Ehlers and Clark's (2000) cognitive theory of PTSD proposed that symptoms persist only if individuals process the trauma in a way that results in a sense of on-going threat. They suggested that this threat might be the product of other emotions as well as fear, including shame, identified as damaged self-concept. Lee, Scragg, and Turner (2001) proposed a clinical model of shame and guilt-based PTSD, suggesting that shame can be seen as a current threat in that it attacks the person's psychological integrity, resulting in feelings of inferiority, social unattractiveness and powerlessness. In a summary of recent research on the psychological processes implicated in PTSD, Brewin and Holmes (2003) found that posttrauma appraisal frequently resulted in increased negative emotions, including shame, and associated these with slower recovery from PTSD.

## Intrapersonal and interpersonal shame

Shame can operate at the level of the individual, the interpersonal relationship, the group, and even the culture (Gilbert & Andrews, 1998) and can be generated in a number of ways. At the level of the individual, intrapersonal shame can have an internal or an external origin (Gilbert, 2001), or be a combination of both (Cook, 1996). Internal shame may be associated with a belief that one has not lived up to one's personal value system in the context of one's thoughts, behaviour, or appearance. It results in a devaluation of one's self-concept—a belief that one is less worthy, less capable, weak, or inadequate. External shame involves the judgements of others which, if accepted and internalised, become internal shame as well (Gilbert & Andrews, 1998). An example of how internal shame might occur, but be difficult to identify, might be the soldier or police officer who kills in the line of duty. Their behaviour might be considered entirely appropriate and justified in their terms of engagement or training, but completely antithetical to their personal underlying values, and hence destructive to their self-concept. Also at the intrapersonal level, Janoff-Bulman (1992) and Edmondson et al. (2011) proposed that trauma can shatter deeply held core beliefs or assumptions about personal identity and the nature of the world, resulting in a sense of powerlessness and resultant shame and fear. These shattered beliefs and assumptions have in turn been linked to changes in self-identity or self-concept, thought to represent an important, largely unexamined factor in the pathogenesis of PTSD (Berntsen & Rubin, 2006; Ehlers & Clark, 2000).

At a close interpersonal level, shame is considered a signal of risk to the social bond—a sign that the other might be disapproving of one's actions or self, and that this might culminate in rejection (Scheff, 2000). In a study linking partner emotional support, negative interaction and trauma, Cox, Buhr, Owen, and Davidson (2015) found that emotional support was linked to reduced distress, while negative interaction was strongly linked to increased distress. A similar result was found by Robinaugh et al. (2011), who found that negative dyadic interaction was associated with the maintenance of PTSD symptom severity because of its association with negative posttrauma cognitions.

At the more distant interpersonal level, shame can be evoked by loss of status, as in losing one's standing in a social group or one's job, or letting one's family or team down (Gilbert, McEwan, Bellew, Mills, & Gale, 2009). Loss of social rank was found to be predictive of a diagnosis of PTSD in a study investigating “mental defeat” (Troop & Hiskey, 2013). In the culture of the military or emergency services being responsible for, or having been unable to prevent, injury or loss of life can be a source of shame (Lifton, 1993), as can simply having survived when others did not (Wilson, Drozdek, & Turkovic, 2006). In a larger societal sense, veterans returning home from the unpopular war in Vietnam were frequently subjected to public shaming and stigma (Herman, 1992). At a cultural level, Scheff (1994, 1997) has related the upsurge of world terrorism to the shame and humiliation experienced by disenfranchised and disempowered honour cultures and religious groups.

As an example of how shame might operate at multiple levels, a police officer who vomited at an incident involving scattered body parts and suffered shame at the time as a result of perceiving himself or herself unable to cope adequately, would experience primary, internal shame. Secondary internal shame might then occur as the police officer reflected on this event and increasingly doubted their ability to cope adequately with future similar incidents. Secondary external shame might also be present if they lose status in the eyes of their peers and become objects of criticism or ridicule. The primary internal shame might form the content for intrusions related to the incident, and this and the secondary—internal and external—shame might then intensify and culminate in increased symptoms or a co-morbid disorder.

## Shame: adaptive and maladaptive presentations

In common with all primary affects, shame has the potential to function adaptively, playing a constructive role in social interaction. Through ordinary experiences of shame, which include self-consciousness, embarrassment, or feeling foolish, individuals are thought to learn boundaries for socially acceptable behaviour (Retzinger, 1995). Because they do not consider it a “negative” affect *per se*, Elison (2005) and Webb (2003, 2010) suggest subsequent psychopathology results from maladaptive shame regulation strategies. Shame then remains unacknowledged, and is expressed as another affect or combination of affects, or as avoidant behaviour.

A number of different theorists relate shame to the emergence of other symptoms. From a background of psychoanalytic theory, Lansky (2000) described how shame remains hidden from awareness following trauma. He identified an altered ego state, disorganised and at risk of fragmentation, that he called the “posttraumatic state.” He proposed that this state gives rise to shame as a result of the person believing they no longer meet their ego ideal or belief in their prior identity. In addition, because this state is disempowering and frightening, he suggested it results in defences that keep shame-arousing awareness from consciousness, and replace it with a variety of pathological phenomena which may include impulsive self-destructive behaviours, withdrawal, or anger. He saw this as a defence against the sense of fragility, neediness, and resultant shame that invariably accompanies the posttrauma state. H. B. Lewis (1971) described shame as a “sleeper” in psychopathology because of its many disguised presentations, where shame can be “unacknowledged” or “bypassed,” resulting in the emergence of other affects or behaviours.

Theorists who have connected shame with anger include Scheff (2011) and Gilligan (1997, 2001) who maintained that *all* violence has some form of bypassed shame at its core. They consider that disrespect from others is experienced as shame/humiliation and retributive aggression results from this. Elison et al. (2014) conceptualised shame as social pain—the pain of feeling unvalued or excluded—with the response of anger and violence as a maladaptive defence. Tangney and Dearing (2002) related the shame response to the emergence of anger and aggression and used this as confirmation of their belief that shame is a destructive emotion. Scheff (2014) described the phenomenon of the recursive “feeling trap” to explain how the emotions might persist over time. Applied to shame, he proposed that one can become ashamed because one is ashamed, or angry because one is ashamed, then ashamed because one is angry, and so on, gathering increasing force with time, and potentially leading to depression or self-harm. It is possible also that shame diverted into anger, combined with the hyperarousal features of the disorder, could account for the frequency of anger reactions in PTSD, as described by McHugh, Forbes, Bates, Hopwood, and Creamer (2012). If this is so, anger management techniques as they are employed in the presence of PTSD (and perhaps in a more general sense) might require an examination of the possible presence of underlying shame as the driver of the anger, as proposed by Velotti et al. (2014).

**Fig. 1:**
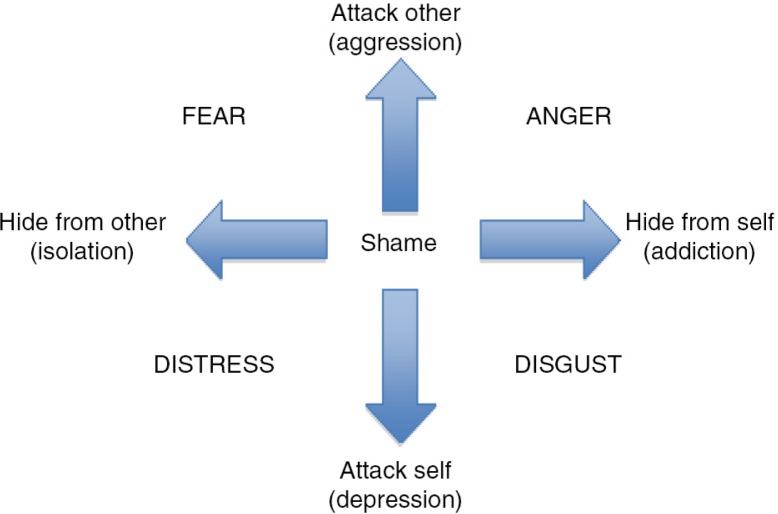
Compass of shame-avoidant behaviours and masking emotions (Webb, 2010, developed from Nathanson, 1992).

Scheff (1994) described shame as the “master emotion” with a central role in evoking a range of other emotions. Nathanson (1987, 1992) similarly conceptualised shame as a key emotion, proposing a “Compass of Shame,” with shame in a central position and shame-related behaviours summarised as: “attack other,” “attack self,” “withdrawal,” and “avoidance.” The theory behind the Compass of Shame suggested that individuals develop scripts or schemas in order to ignore, reduce, or displace shame, without directly addressing its origin. Webb (2003, 2010) proposed developments to this concept, suggesting that the behaviours map a compass of shame-avoidance rather than shame itself. He adopted the language used by participants in a qualitative study to rename Nathanson's (1987) “withdrawal” and “avoidance” poles as “hide from other” and “hide from self,” thereby more clearly identifying the bi-polar dimensions of aggression and alienation of the basic shame-avoidant responses. The four poles thus correspond to the social behaviours of “aggression,” “depression,” “isolation,” and “addiction,” together with their associated avoidant emotions of fear, anger, distress, and disgust (see Fig. 1).

This theory was lent empirical support by Elison, Lennon, and Poulos (2006) and Elison, Poulos, and Lennon (2006), who developed a Compass of Shame Scale. This found supportive evidence for the four distinctive shame-related behaviours. The authors concluded that each approach could be adaptive or maladaptive, depending on the context. The four types of shame-avoidant behaviours bear a strong resemblance to the prominent symptoms and behaviours associated with PTSD.

## Shame and fear: neurobiological aspects

In the sense that shame may signal a threat to sense of self, perception of the world as a safe and predictable place, close relationships and/or standing in a group, it is understandable that it would serve to activate neural mechanisms associated with fear. In the case of the individual exposed to fear evoking trauma, this would have the effect of feeding in to an already activated fear response. A mechanism whereby this might occur is described by Lanius, Frewen, Vermetten, and Yehuda (2010), who proposed two pathways to PTSD following trauma: fear conditioning and early life vulnerabilities. In the fear conditioning model, progressive augmentation of the fear response is thought to occur through repeated exposure to stimuli evoking the emotion, resulting in increasing strengthening of the response and its expansion into neighbouring neural circuits. These include the ventromedial prefrontal cortex, amygdala, and hippocampus. Because this progressive augmentation occurs over time, it provides a model for the frequency of delayed presentation of PTSD. Diagnosis of the disorder more than 6 months after exposure to trauma following exacerbation or reactivation of previous symptoms was found to represent over one third of military and over 15% of civilian cases in studies reviewed by Andrews, Brewin, Philpott, and Stewart (2007).

The second pathway to PTSD included the effects of early childhood environment. Lanius et al. (2010) suggested that disorders of attachment, which may, but do not necessarily include maltreatment or abuse, could play a part in a reduced ability to regulate emotions through dysfunctional development of the emotional and arousal regulating systems. This leads to impaired ability to regulate physiological arousal to threat and vulnerability to trauma-related disorders. The process of emotion and arousal regulation has been defined by Frewen and Lanius (2006) as a medial–frontal/paralimbic modulation of lower level systems of emotional responses to incoming stimuli. This is considered to involve a range of affects clinically prominent in PTSD including shame, in addition to fear. Arousal may be undermodulated, resulting in re-experiencing and hyperarousal, or overmodulated, leading to dissociative symptoms.

An association between attachment disorders and PTSD was supported in a longitudinal study of the relationship between mothers with a diagnosis of PTSD and their infants by Enlow, Egeland, Carlson, Blood, and Wright (2014), who found that an insecure mother–infant attachment relationship increased the risk of developing PTSD following trauma exposure at age 17.5 years. Because shame signals a threat to the social bond, which is manifest in its primary form in mother–infant attachment, it is likely that early disruption in this relationship could provide later increased vulnerability to shame associated with perceived loss of support and/or damaged self-concept following trauma.

Lowered cortisol leading to a prolonged stress response has been consistently found in many individuals with a diagnosis of PTSD and is considered to have its origins in childhood adversity and present a vulnerability to PTSD following later exposure to trauma (Yehuda & Seckl, 2011). In an investigation of the psychosocial factors associated with low cortisol, Mason et al. (2001) found the most prominent factors to be disengagement and shame laden depression, which they related to inconspicuous but potentially overwhelming shame resulting from both primary and secondary traumatisations.

## The importance of shame in treatment and management

Because the perception of shame in others can also evoke a discomforting emotion in the observer, it may fail to be addressed in therapy (Lansky, 2003; H. B. Lewis, 1971). Shame may remain unidentified in an unconscious collusion with the patient to fail to recognise the distressing emotions (Wilson et al., 2006).

The aspect of shame that involves stigma associated with seeking treatment is well documented, particularly in the case of military or police, whose training and sub-culture emphasise stoicism (Hoge, 2010). Less well recognised is the shame that may be present in the therapeutic situation itself when the patient is required to display his or her vulnerability to the therapist, who might be perceived as impatient, judgemental, or disinterested (Lazare, 1987).

Most theoretical accounts of PTSD have emphasised fear as the primary emotion associated with this disorder. However, other affects, including shame, may also form part of the response, but may not be readily volunteered. Shame associated with a traumatic event was found to impede emotional processing (Brewin, Dalgleish, & Joseph, 1996), impact negatively on the therapeutic alliance (Black, Curran, & Dyer, 2013), and potentially serve to worsen the posttrauma reactions in the context of treatment because it has the potential to contribute to later psychopathology and affect help-seeking (Lee et al., 2001). In light of these findings, the recommendation that issues of shame associated with changes in self-concept be addressed in as an integral part of treatment (Ford, Courtois, Steele, van der Hart, & Nijenhuis, 2005) might usefully be adopted. Changes in close relationships, occupation or social standing, and associated shame are also suggested as important areas for initial investigation.

Unacknowledged shame and its diversion into shame-avoidant behaviours is a further significant area for examination, not only because it masks awareness of the underlying emotion and hence affects treatment, but also because it may further exacerbate symptoms. Shame-avoidant anger may, for example, evoke further shame as the individual reflects on its effect on others, and the changes to their own self-image. It is likely that the military and police, in particular, are prone to this aspect of shame-avoidant emotion and related behaviour, since their training emphasises defensiveness in the face of threat (Hoge, 2010).

## Conclusions and implications for treatment

DSM-5 has given us a diagnosis of PTSD based on its phenomenological presentation, which includes the persistent emotional state of shame. This article addresses the underlying dynamics of this emotion and its complex interactions with other emotions and behaviours and proposes that it might function as a unifying and core component in the exacerbation, maintenance and delayed presentation of PTSD, and the development of co-morbidities.

However, because recognition of shame in itself is often considered shaming, identifying the extent of its presence in the initial stages of therapy may be problematic. It might best be accomplished through examination of three potential domains: *intrapersonal shame*, the examination of any changes in self-concept; *interpersonal shame at the intimate level*, which might include any changes in personal relationships; and *interpersonal shame at an occupational and societal level*, which could include issues of loss, isolation, and exclusion.

Treatment might then usefully explore these changes in terms of the “sleeper” of unacknowledged shame—how initial shame signals might have been diverted into maladaptive shame regulation strategies; how these have contributed to the manifestation of symptoms of the posttraumatic state; and how they might be addressed by more shame-adaptive responses that reconstruct and reinforce positive self-identity and significant personal and social relationships.
